# Case report: *Francisella philomiragia* bacteremia in a patient with acute lymphoblastic leukemia

**DOI:** 10.3389/fcimb.2023.1206972

**Published:** 2023-09-14

**Authors:** Dee Xi

**Affiliations:** Department of Clinical Laboratory, The First College of Clinical Medical Science, China Three Gorges University, Yichang Central People’s Hospital, Yichang, China

**Keywords:** *Francisella philomiragia*, acute lymphoblastic leukemia, 16S rRNA, blood culture, bacteremia, antibiotics

## Abstract

*Francisella philomiragia* is a Gram-negative coccobacillus, which is a very rare human opportunistic pathogen causing pneumonia and systemic infection. It is difficult to identify this bacterium through conventional Gram-staining and biochemical methods due to an amorphous Gram stain appearance after 24 h culture and its relatively fastidious and slow growth giving weak and/or delayed reactions in biochemical tests. It is often misidentified as other bacteria including *Haemophilus* spp., *Pseudomonas aeruginosa*, or *Sphingomonas paucimobilis.* False identification may delay the therapy of the patients and even endanger the patient’s life. Here, we report a case of a 34-year-old man with acute lymphoblastic leukemia infected by *F. philomiragia*, which was almost misdiagnosed. This case describes our identification of a patient with a systemic *F. philomiragia* infection. To our knowledge, this is the first such case reported in China.

## 1 Introduction

*Francisella philomiragia* is a strictly aerobic, Gram-negative coccobacillus, which is a very rare human opportunistic pathogen. There are just 22 human infected cases reported around the world until now (Wenger et al., 1989; Sicherer et al., 1997; Friis-Møller et al., 2004; Mailman and Schmidt, 2005; Kreitmann et al., 2015; Relich et al., 2015; Robles-Marhuenda et al., 2018; Froböse et al., 2020; Chua et al., 2021). *F. philomiragia* infection historically has been reported in patients with underlying conditions including CGD, hematological malignancy, or near-drowning events (Wenger et al., 1989; Sicherer et al., 1997; Friis-Møller et al., 2004; Mailman and Schmidt, 2005; Relich et al., 2015; Robles-Marhuenda et al., 2018; Froböse et al., 2020). As a member of the *Francisella* genus, *F. philomiragia* is an opportunistic pathogen causing fever, pneumonia, and systemic infection such as bacteremia. Different from its more virulent relative *Francisella tularensis*, *F. philomiragia* infection related to saltwater exposure usually occurs in immunocompromised or near-drowning people (Wenger et al., 1989; Sicherer et al., 1997; Friis-Møller et al., 2004; Mailman and Schmidt, 2005; Kreitmann et al., 2015; Relich et al., 2015; Robles-Marhuenda et al., 2018; Froböse et al., 2020; Chua et al., 2021) while tularemia transmitted by the bite of arthropods, contacting infected animals, consuming contaminated water and food, or inhalation of contaminated aerosols (Fooladfar and Moradi, 2023) always occurs in people with normal immunity apart from a few cases with abnormal immunity (Wenger et al., 1989). Confusion with *F. tularensis* may lead to false biohazard alarms. *F. tularensis* is a Risk Group 3 pathogen and a potential biological weapon (Mailman and Schmidt, 2005) because of its strong virulence and easy diffusion through aerosol, and because it exists in the environment for a long time (Wenger et al., 1989; Mailman and Schmidt, 2005; Freudenberger Catanzaro and Inzana, 2020), potentially leading to high numbers of secondary tularemia cases. Limited awareness of *F. philomiragia* has caused delayed diagnosis, therapeutic failures, and bad outcomes. Here, we present the first case of *F. philomiragia* infection reported in China. A 34-year-old man with acute lymphoblastic leukemia was infected by *F. philomiragia* after 16 days’ chemotherapy.

## 2 Case presentation

**Figure 1:**
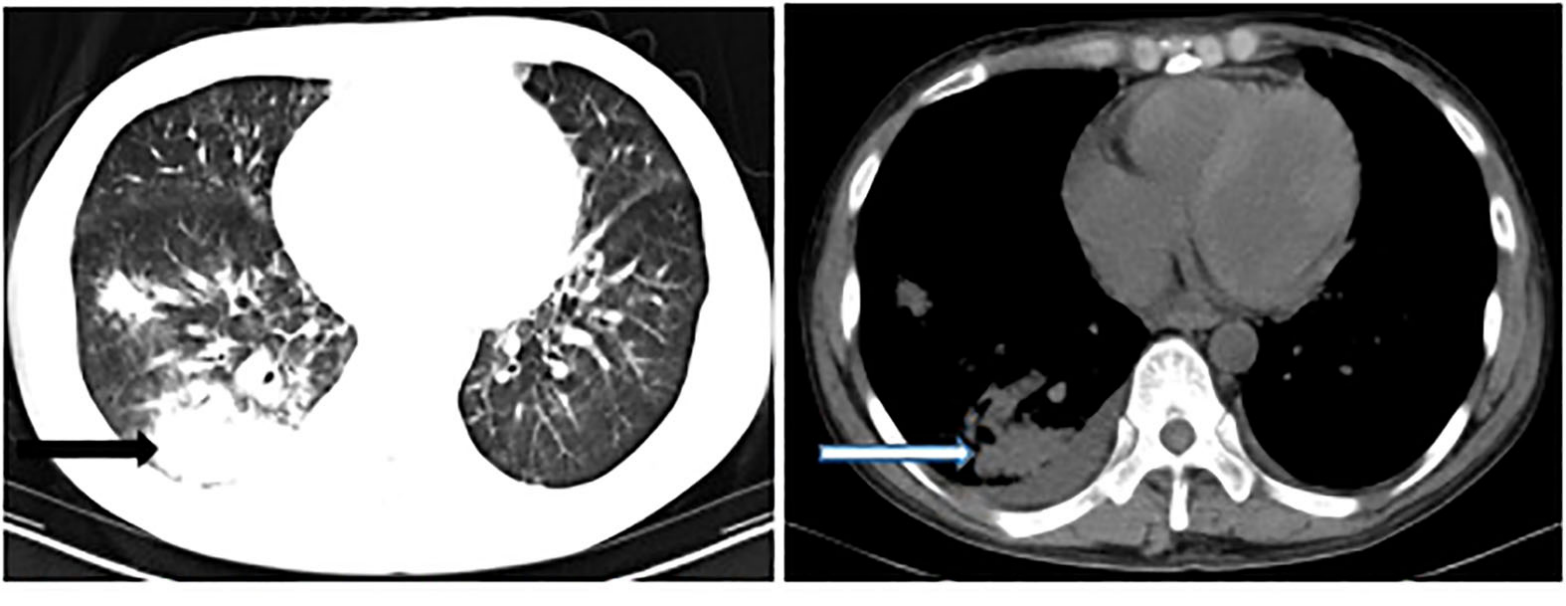
Computed tomography examination of the lungs showed multiple patchy shadows, especially in the right lung (arrow).

**Figure 2:**
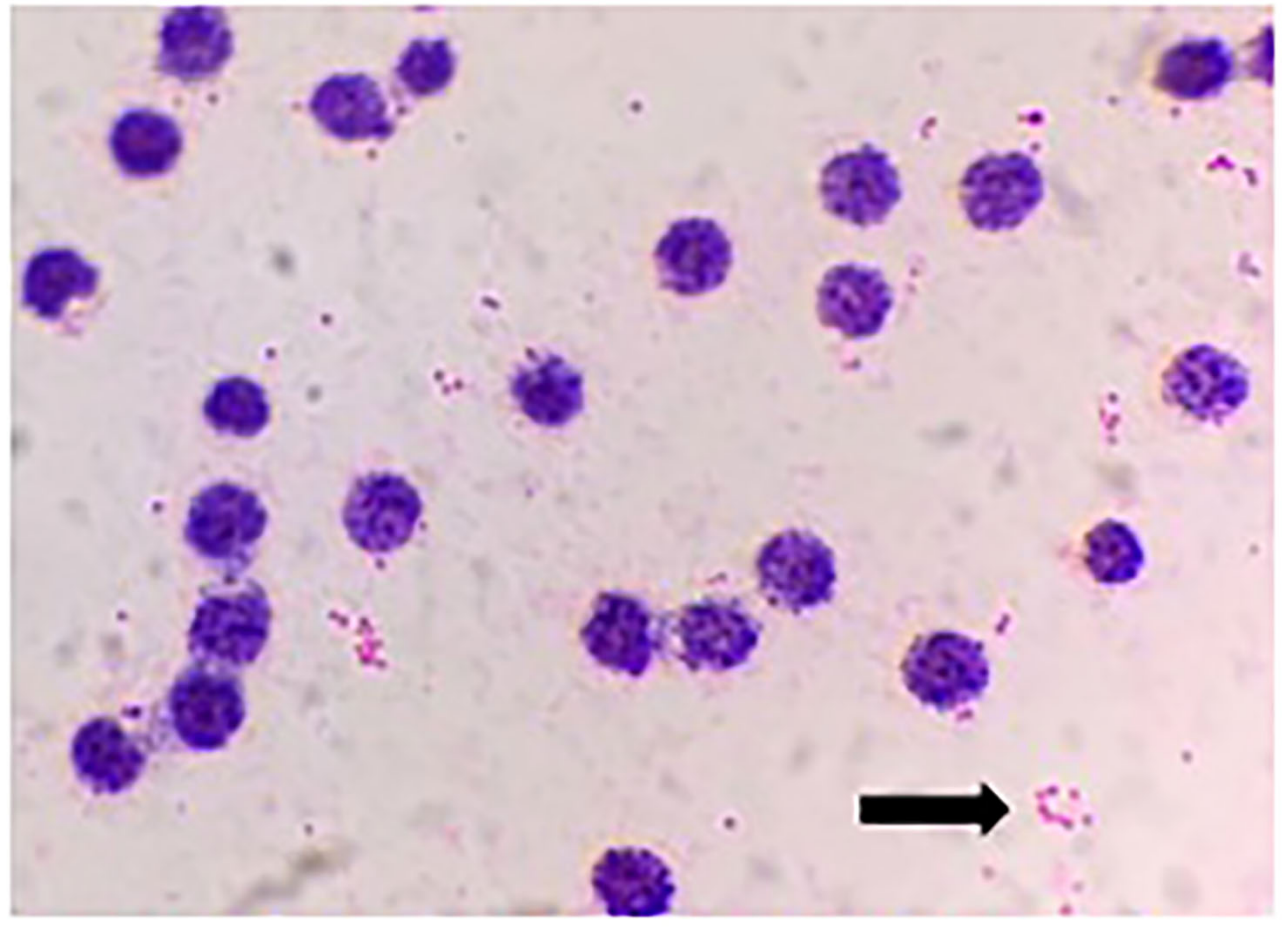
Gram stain of blood culture both showed Gram-negative coccobacilli (arrow).

**Figure 3:**
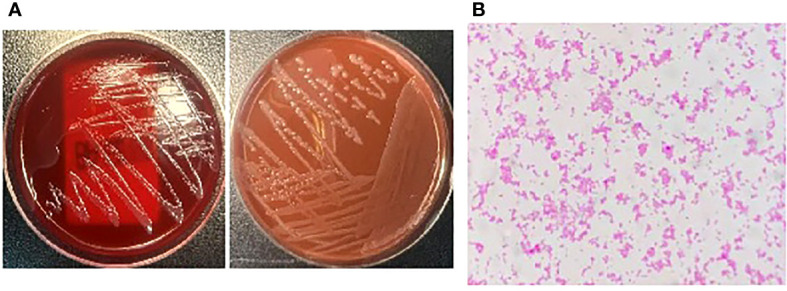
The bacteria was incubated in the incubator at 35°C in 5% CO2 for 24h, **(A)** The plates grew medium-sized (diameter about 5mm), glossy, convex colonies. **(B)** Gram stain of the single colony showed Gram-negative coccobacilli.

A 34-year-old man was admitted to a local hospital because of fever and fatigue. The results of a routine blood test showed that the patient had a high white blood cell count (WBC; 16.4 × 10^9^/L) and a very high percentage of immature granular cells (72%), and a low red blood cell count (RBC; 2.42 × 10^12^/L) and a low blood platelet count (PLT; 16 × 10^9^/L). Then, the patient was given other examinations including bone marrow hemocytology (original cells 73%, and peroxidase staining <3%), cellular immunology (flow cytometry detected 82% of abnormal cells expressing the following biomarkers: HLA-DR, CD10, CD19, CD20, CD22, CD34, CD38, CD58, CD123, CD79a, and TdT), and gene detection [BCR/ABL1(P190) fusion gene was positive]. On the basis of these results, the patient was diagnosed with acute lymphoblastic leukemia and was admitted to our hospital. Chemotherapy with Dasatinib plus DVP was started 2 days after his admission. After 16 days’ chemotherapy, the patient complained of chest tightness, shortness of breath, dyspnea, cough and expectoration, blood in phlegm, edema of both lower extremities, weakness of limbs, and fever with a maximum body temperature of 39.2°C. Physical examination revealed moist crackles in both lower lungs, obvious on the right side, and the blood oxygen saturation decreased to 86%. The empirical treatment with antibacterial cefoperazone-sulbactam and antifungal fluconazole was initiated. Computed tomography examination of the lungs showed multiple patchy shadows in both lungs, mainly in the lower right lung, with partial consolidation and bilateral pleural effusion (**Figure 1**). Blood routine test revealed the following levels: leukocyte, 0.36 × 10^9^ cells/L; erythrocyte, 1.96 × 10^12^ cells/L; hemoglobin, 61 g/L; platelets, 46 × 10^9^ cells/L; and neutrophil, 0.26 × 10^9^ cells/L. Elevated levels of procalcitonin (0.211 ng/ml) and CRP (54.23 mg/L) were also noted. Four bottles of blood culture samples were drawn at once. One of the aerobic bottles of blood cultures signaled positively after approximately 22.2 h of incubation in the blood culture instrument (BacT/Alert 3D, Biomérieux, France). Gram stain of broth from this bottle showed Gram-negative coccobacilli (**Figure 2**). The blood culture broth was subcultured with a blood and a chocolate agar plate. Medium-sized (diameter approximately 5 mm), glossy, convex colonies grew on the plates (**Figure 3A**) after 24 h of incubation in the incubator at 35°C in 5% CO_2_. Gram stain of the single colony showed Gram-negative coccobacilli (**Figure 3B**), the same as those seen in the blood culture smear. The bacteria were identified as *Sphingomonas paucimobilis* by the automatic microbial identification and antimicrobial susceptibility testing system (Vitek 2 Compact, Biomérieux, France). The mass spectrometer (Vitek MS, Biomérieux, France) did not yield an identification result. The discrepancy in identification between the Vitek 2 Compact and mass spectrometer data might have reflected underlying differences in the databases associated with each testing type, and thus, further testing to identify the bacterium in the blood culture was undertaken. 16S rRNA gene sequencing identified the pathogen as *F. philomiragia*. At the same time, NGS (next-generation sequencing) (MGISEQ-200, BGI, China) detected *F. philomiragia* and *Legionella* in the bronchoalveolar lavage fluid of the patient. After the bacteria were identified, the clinician replaced the antibiotics with moxifloxacin and azithromycin. The patient’s symptoms gradually improved.

## 3 Discussion

*F. philomiragia* is an intracellular Gram-negative non-motile coccobacillus (Propst et al., 2016), belonging to the *Gammaproteobacteria* class and *Francisellaceae* family (John et al., 2019). *F. philomiragia* seems to like aquatic environments as it has been found near bodies of water, particularly brackish or saltwater in the mainland United States (Hollis et al., 1989; Berrada and Telford, 2010; Siddaramappa et al., 2012) and coastal waters in Norway (Duodu et al., 2012). *F. philomiragia* is a very rare human opportunistic pathogen, which has been isolated from water, muskrats, dogs, and humans (Hollis et al., 1989; Cora et al., 2010). *F. philomiragia* infection mainly occurs in immunocompromised patients, such as CGD patients who fail to produce superoxide anion and other oxygen metabolites including H_2_O_2_ to kill catalase-producing microorganisms (e.g., *Staphylococcus aureus*, *Pseudomonas aeruginosa*, enteric bacilli, and *Aspergillus* spp.) because of defective oxidase function in the circulating phagocytes (Wenger et al., 1989; Friis-Møller et al., 2004), patients with hematologic malignancies who receive long-term chemotherapy drugs (Kreitmann et al., 2015), and the patient with polycystic kidney receiving long-term immunosuppressive agents after renal transplantation (Relich et al., 2015). Exposure to seawater is also more prone to be infected by *F. philomiragia*. Some CGD patients had *F. philomiragia* infection after a seaside vacation (Friis-Møller et al., 2004; Robles-Marhuenda et al., 2018; Froböse et al., 2020). A 10-year-old CGD patient was infected by *F. philomiragia* because of a facial abrasion caused by a seawater crab tossed by the patient’s friend (Mailman and Schmidt, 2005). The most common symptoms of *F. philomiragia* infection are fever and pneumonia (Wenger et al., 1989; Friis-Møller et al., 2004; Mailman and Schmidt, 2005; Kreitmann et al., 2015; Relich et al., 2015; Robles-Marhuenda et al., 2018; Froböse et al., 2020; Chua et al., 2021), and meningitis and peritonitis have also been reported (Relich et al., 2015; Robles-Marhuenda et al., 2018). Deaths due to sepsis have also been reported (Fooladfar and Moradi, 2023). It was found that *F. philomiragia* could infect healthy lung tissue via alveolar macrophages or epithelial cells, which explains why pneumonia is the common symptom after *F. philomiragia* infection in patients (Propst et al., 2016). Generally speaking, patients are prone to be infected by *F. philomiragia* through saltwater exposure. Some reports have suggested infection via aerosols or insect bites, although these claims are not well-supported (Kreitmann et al., 2015). In this case, the patient became infected during hospitalization. However, the patient had gone to the city near a sea before admission; it was possible that he had contact with seawater or a marine organism, which was a possible source of infection. *F. philomiragia* and *Legionella* were both detected in the bronchoalveolar lavage fluid of the patient, which would suggest the patient may have been infected by exposure to seawater/aerosols contaminated by both bacterial species; a case like this has never been reported before. *F. philomiragia* was found not only in blood but also in the bronchoalveolar lavage fluid of the patient, which would suggest a possible respiratory source for the bacteremia.

Incubated with aquatic amoebae, *F. philomiragia* can form robust biofilms to exist in water for a long time (Verhoeven et al., 2010). That may explain the prevalence of *F. philomiragia* infection in patients contacting seawater and marine organisms. Furthermore, one study showed that *F. philomiragia* was able to infect the human hepatocyte-like cell line HepG2 cells. This finding suggested that *F. philomiragia* might replicate in the liver of near-drowning victims, so the sequelae of infected patients should be closely observed (Propst et al., 2016).

Originally assigned to the genus *Yersinia*, *F. philomiragia* is closely related to *F. tularensis* (Relich et al., 2015), a tier 1 select agent (Freudenberger Catanzaro and Inzana, 2020), and confusion in identification might lead to unnecessary biohazard alarms (Mailman and Schmidt, 2005). As there are differences in biochemical reactions between *F. philomiragia* and *F. tularensis* (**Table 1**), they can be readily distinguished from one another. *F. philomiragia* is frequently detected in the blood of patients (Wenger et al., 1989; Friis-Møller et al., 2004; Kreitmann et al., 2015; Relich et al., 2015; Robles-Marhuenda et al., 2018; Chua et al., 2021), and is also found in lymph node tissue (Mailman and Schmidt, 2005; Chua et al., 2021), lung biopsy, cerebrospinal fluid, and thoracoabdominal fluid (Wenger et al., 1989), while the detection in sputum and alveolar lavage fluid is uncommon.

**Table 1:** Biochemical differences between *F. philomiragia* and *F. tularensis*.

Tests	*F. philomiragia*	*F. tularensis*
Oxidase, Kovacs	Positive	Negative
H_2_S production in triple sugar iron agar	Positive	Negative
Gelatin hydrolysis	Positive (80%)	Negative
Catalase	Positive or weakly positive	Weakly positive

It is not easy to obtain accurate identification of *F. philomiragia.* On the one hand, Gram staining shows very small coccobacillus with diverse morphology, fine sand-like scattered distribution, and *F. philomiragia* may be even mistaken for impurities in the background of blood culture smear to be neglected. Repeated isolation of cultures on solid medium made *F. philomiragia* more visible with Gram staining due to their small ball and rod-shaped morphology (Friis-Møller et al., 2004). On the other hand, difficulty exists in conventional biochemical identification of the isolate probably due to its relatively fastidious nature with slow growth giving weak and delayed reactions, so it is easy to identify *F. philomiragia* as other bacteria (Relich et al., 2015). In this report, *F. philomiragia* was wrongly identified as *S. paucimobilis* by GN card (Vitek 2 GN, Biomérieux, France). Additionally, the mass spectrometer (Vitek MS, Biomérieux, France) did not yield an identification result because this bacterium was not in the database of the instrument. However, *F. philomiragia* identified by the mass spectrometer (Bruker Daltonics, Bremen, Germany) has been reported, and the results were in accordance with that of gas chromatography and 16S rRNA gene sequencing (Froböse et al., 2020). Some mass spectrometers cannot identify *F. philomiragia* probably because this bacterium is not within their databases, and the identification becomes possible when adding *F. philomiragia* spectra in the database. The identification of *F. philomiragia* needs to be confirmed by 16S rRNA gene sequencing (Friis-Møller et al., 2004; Mailman and Schmidt, 2005; Robles-Marhuenda et al., 2018; Froböse et al., 2020). *F. philomiragia* is very rare in daily practice, which is not well known by us, so it may be neglected or identified by mistake. With the development of advanced technologies in molecular biology and more knowledge about this bacterium, it will become easier to identify *F. philomiragia.*

In clinical practice, if you encounter the very small Gram-negative coccobacillus with unusual morphology just like that in **Figures 2**, **3B**, which was identified as *S. paucimobilis* or other bacteria by GN card, you must be particularly careful. In order to ensure the accuracy of the identification, it is best to perform 16S rRNA gene sequencing to confirm the result. False identification may lead to incorrect treatment, which will delay the patient’s disease course and may even lead to death in severe cases.

To date, there is no standardized guideline for the treatment of *F. philomiragia*. Hollis et al. found that the isolates of *F. philomiragia* were sensitive to aminoglycosides, tetracycline, chloramphenicol, cefoxitin, and cefotaxime but were resistant to ampicillin; 79% of the isolates were moderately sensitive to erythromycin, and all produced β-lactamase (Wenger et al., 1989). Robles-Marhuenda et al. found that most of the isolates were susceptible to quinolones, chloramphenicol, and carbapenems but resistant to ampicillin, and all produced β-lactamase. Third-generation cephalosporins, aminoglycosides, and tetracyclines may also be useful (Robles-Marhuenda et al., 2018). Froböse et al. found that the isolate of *F. philomiragia* was resistant to trimethoprim/sulfamethoxazole and sensitive to meropenem and ciprofloxacin (Froböse et al., 2020). Antimicrobial susceptibility testing with the human antimicrobial peptide LL-37 and the mouse antimicrobial peptide mCRAMP showed that these peptides were highly active against *F. philomiragia* and could kill bacteria *in vitro*. However, the antimicrobial peptide host defense mechanism was clearly not sufficient to control infected cells or *F. philomiragia* infection *in vivo* (Propst et al., 2016). The treatment of *F. philomiragia* varies greatly, mainly depending on the patient’s disease status and underlying conditions. Among the previously reported cases of *F. philomiragia* infection, one patient with fever and pleuritis was treated without antibiotics, and in the other cases, the patients were given two to four antibiotics (Relich et al., 2015).

## Data availability statement

The raw data supporting the conclusions of this article will be made available by the authors, without undue reservation.

## Ethics statement

Informed consent was obtained from all subjects involved in the study. Written informed consent was obtained from the participant/patient(s) for the publication of this case report.

## Author contributions

The author confirms being the sole contributor of this work and has approved it for publication.
